# Photothermal Microscopy and Spectroscopy with Nanomechanical Resonators

**DOI:** 10.1021/acs.jpcc.3c04361

**Published:** 2023-11-06

**Authors:** Robert G. West, Kostas Kanellopulos, Silvan Schmid

**Affiliations:** Institute of Sensor and Actuator Systems, TU Wien, Gusshausstrasse 27-29, 1040 Vienna, Austria

## Abstract

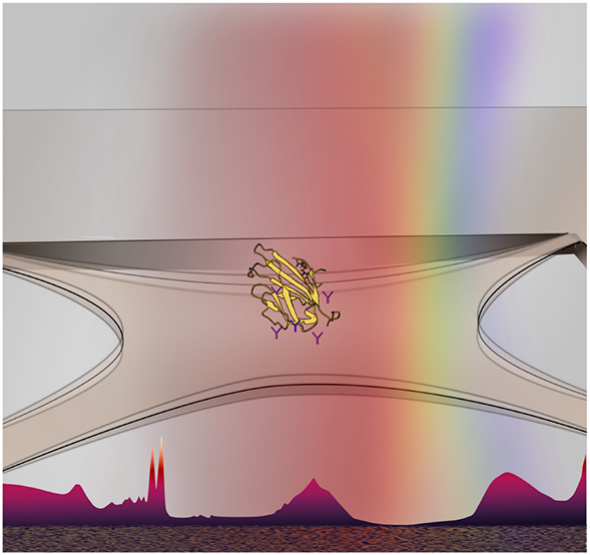

In nanomechanical photothermal absorption spectroscopy and microscopy, the measured substance becomes a part of the detection system itself, inducing a nanomechanical resonance frequency shift upon thermal relaxation. Suspended, nanometer-thin ceramic or 2D material resonators are innately highly sensitive thermal detectors of localized heat exchanges from substances on their surface or integrated into the resonator itself. Consequently, the combined nanoresonator-analyte system is a self-measuring spectrometer and microscope responding to a substance’s transfer of heat over the entire spectrum for which it absorbs, according to the intensity it experiences. Limited by their own thermostatistical fluctuation phenomena, nanoresonators have demonstrated sufficient sensitivity for measuring trace analyte as well as single particles and molecules with incoherent light or focused and wide-field coherent light. They are versatile in their design, support various sampling methods—potentially including hydrated sample encapsulation—and hyphenation with other spectroscopic methods, and are capable in a wide range of applications including fingerprinting, separation science, and surface sciences.

## Introduction

Absorption spectroscopy, a tool for identifying species and investigating molecular and nanoparticle properties, is traditionally performed at concentrations suitable for the sensitivity of ensemble measurements. In standard applications, such measurements often mask the intrinsic responses of individual molecules due to the variability in degrees of freedom, isomerization, and environmental interactions. The heterogeneity in size, shape, and composition of particles in samples further underscores the need to study absorption responses at the single-molecule and single-nanoparticle level. Advancements in so-called non-flourescence spectroscopy has enabled label-free detection, in most cases, and characterization of scarce concentrations of nanoparticles and molecules.^1−3^ Specifically, developments in photothermal spectroscopy have facilitated the measurement of ever-lessening analyte concentrations as well as subdiffraction-limited localization of a large variety of single nanoparticles and molecules at room and cryogenic temperatures. As a result, photothermal spectroscopy has extended the scope of absorption spectroscopy, offering deeper insights into heterogeneous samples and finding applications in diverse fields like material science, catalysis, nanotechnology, and biophysics.

The field of photothermal spectroscopy incorporates a diverse set of methods based upon the measurement of a substance’s wavelength-dependent absorption by way of its thermal energy transfer to its environment upon relaxation.^1,4−6^ It is a direct measurement of the sample’s absorption via the sample’s own thermal relaxation. Modern photothermal spectroscopy offers notable advantages such as predominantly label-free applications and detected sample absorption free of the impinging light’s scattering background. However, the term ’photothermal’ often refers to techniques which employ a probing light beam to detect the sample’s absorbed heat, induced by a heating beam, based on the resultant optical changes in the surrounding medium.^1,4−6^ In this way, the sample’s immediate environment becomes, in a sense, part of the detector system. However, measuring the probe light requires another detector, such as a photodiode, much further away in free space.

This perspective article addresses a particular fledging method, nanomechanical photothermal spectromicroscopy, which relies on the thermally induced mechanical resonance detuning in nanomechanical resonators. This intimate detection scheme facilitates highly sensitive spatio-chromatic investigations of adhered, adsorbed, or integrated analyte with nanometer to atomically thick mechanical resonators, such as graphene. Not only is the method distinguished by its predisposition to simple optical geometries for probing the entire absorption spectrum of the analyte, it facilitates a diverse range of applications. This perspective delves into these facets, exploring its present and prospective applications where sensitivity limitations are chiefly defined by the intensity fluctuations of a single heating laser and the thermostatistical noise of the resonator. The nanomechanical resonator is emerging as a versatile platform in spectroscopy and microscopy, leaving much to be explored in terms of sample type, signal optimization, scanning and probing methods, and applications.

**Figure 1:**
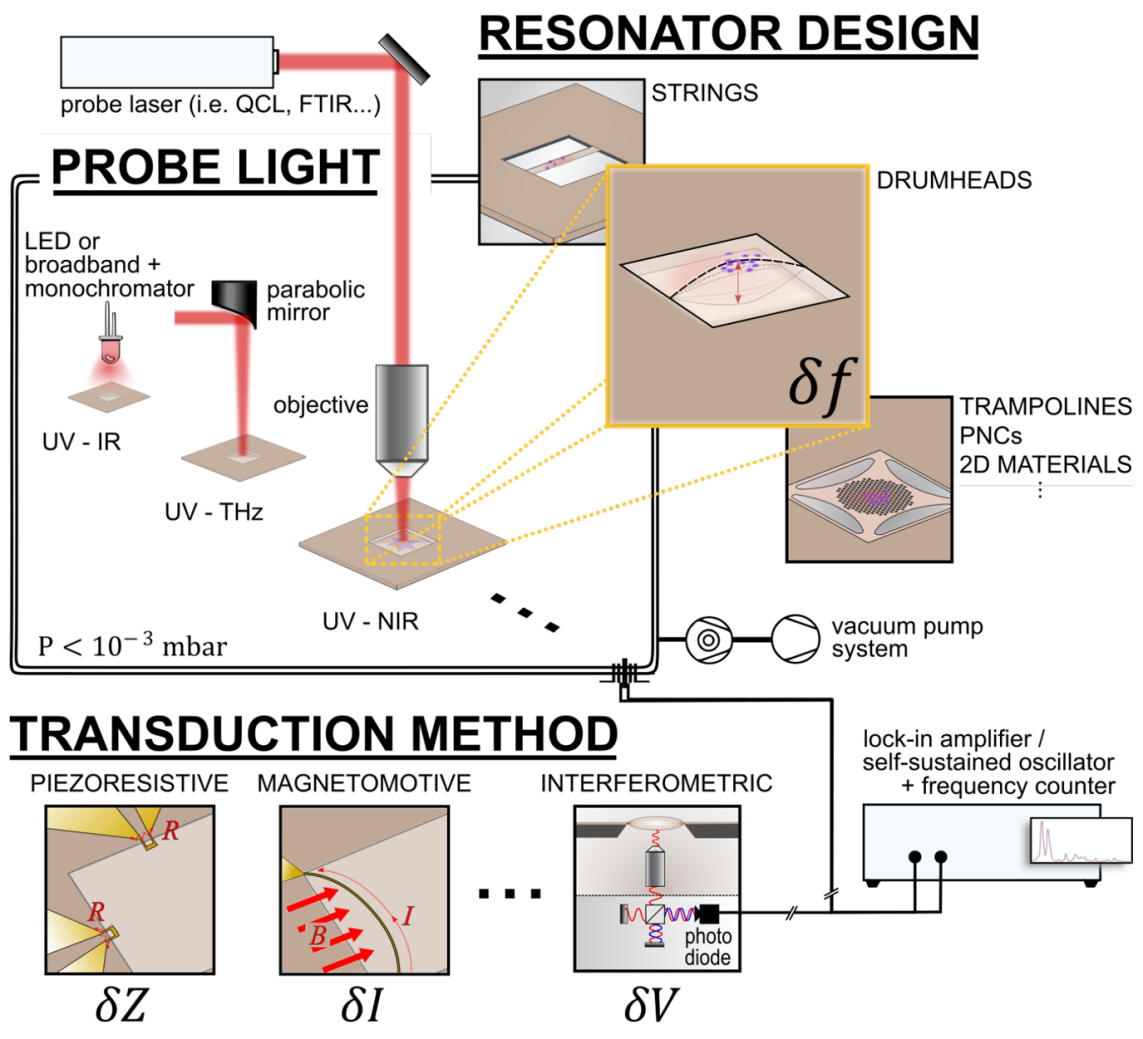
Nanomechanical resonators are highly versatile and adaptable in design and function, to perform highly sensitive broadband absorption spectroscopy for numerous applications in low-pressure environments. They also avail themselves to various means of transduction and frequency-tracking schemes.

The core principle enabling nanomechanical systems to serve as highly sensitive spectrometers is straightforward: light absorbed by the sample, ranging from UV to THz, whether continuous or pulsed, transforms into heat within the thin resonator during thermal relaxation. This heating light induces a frequency shift in the resonator, measured optically by interferometry or electrically, by a variety of methods (see Figure 1). The notion of using stress changes in a resonant structure for spectroscopy dates back to 1969, serving as a basis for designing nanomechanical thermal infrared detectors,^7^ and the intersection of photothermal spectroscopy and microstructures originated with bimaterial cantilevers in the early 1990s. Resonating, nanometer-thick microstructures, such as strings, drumheads, or trampolines, are exceptionally sensitive to local changes in temperature, inducing a change in tensile stress and ultimately limited by fundamental, statistical thermal fluctuations including that described by the fluctuation dissipation theorem.^8,9^ The precise impact and interrelation of these elements within the frequency noise are under active study;^10−13^ however, the extent of heat dissipation and the thermal interfaces in these thin, suspended structures are clearly definable.

Though the nanomechanical resonator and optics-based photothermal methods are rooted in distinct measurement contexts, they each possess their own unique complexities and offer potential areas of mutual application. Optical complexities, such as precise optical overlap, modulation of a heating laser, and optical filtering are requirements often considered synonymously with photothermal spectroscopy. In thermorefractive studies, maintaining the precise focal depth of a probing laser relative to a heating laser is critical, along with the diffraction, screening, and filtering of light.^14^ However, nanomechanical photothermal sensing uniquely alleviates some of these complexities as the substrate, serving as the thermal detector, negates the need for a probing laser, and its high signal-to-noise ratio obviates the need for heating beam modulation. Reducing the amount of optics is also advantageous for studies in the ultraviolet range, where power is generally more significantly attenuated. Nonetheless, it maintains optical versatility, accommodating focused or wide-field, coherent or incoherent light, enabling spectroscopic analysis of diverse entities including thin films,^15,16^ 2D materials,^17,18^ surface-adsorbed chemical species,^19,20^ explosives,^21^ nanoparticle ensembles,^22−24^ and individual single nanoparticles.^25−27^

The simplicity and versatility of the nanomechanical resonator technique afford it several advantages over state-of-the-art mid-IR photothermal techniques.^5,6^ In the context of solid, layered, or adsorbed matter, nanoresonators do not necessitate transparency in the substrate, unlike some optical geometries in other methods, and they function as naturally balanced detectors (see Signal-to-Noise Ratio). The technique requires only the heating beam and vacuum-compatible focusing objectives with high numerical apertures (N.A.) can be used. Likewise, the method avoids the need for spectral filtering because it does not rely upon scattered light, nor are there spectral limitations imposed by some methods with the two-beam geometries. Lastly, as opposed to IR photothermal heterodyne imaging,^5^ the mechanism for transduction of the absorbed power per wavelength in nanomechanics is well-understood and the extent of heating and boundary conditions are clear. Moreover, mechanical resonators provide extensive thermal control, reaching down to the level of single-phonons at cryogenic temperatures,^28^ and precise transduction, even down to below the standard quantum limit for displacement.^29^

Though nanomechanics allow for such impressive versatility, it also lacks the capability of many strictly optical photothermal methods. Contemporary photothermal approaches can be performed with liquid, matrix, supercritical fluid, or even host cell; whereas, the nanomechanical frequency-detuning method must be performed in low pressure environments, typically of <10^–3^ mbar. One important side note, however, is the potential for encapsulation of hydrated samples on suspended silicon nitride, as discussed at the end of this perspective (see Challenges). Likewise, microfabrication methods used to make nanoresonators have also inspired suspended microchannel resonators (SMRs), which enable photothermal resonance-tuning detection in microfluidics. SMRs are capable of single cell and single protein mass detection and have demonstrated sensitivities in hundreds of nJ for absorption spectroscopy.^30,31^ Nevertheless, the low-pressure environment is required in nanomechanics because a vacuum lessens deleterious air damping and convective heat transfer, making it a sensitive detection paradigm for numerous applications, including those exclusive to low-pressure environments.

Other disadvantages include the nanomechanical resonators’ typically longer thermal dissipation times, rendering them less suitable for dynamic studies. Additionally, they cannot perform 2D or 3D imaging, and very high N.A. oil-immersed objectives are only suitable for ambient conditions.^6^ However, they potentially can be combined with other photothermal techniques, depending on the sample and optical geometry. Nonetheless, the central aim of this perspective is to acquaint a broader audience with the advancements and ongoing research in nanomechanical resonance sensing, with a special emphasis on single-molecule absorption spectromicroscopy.

## Advances in Nanomechanical Photothermal Spectroscopy

**Figure 2:**
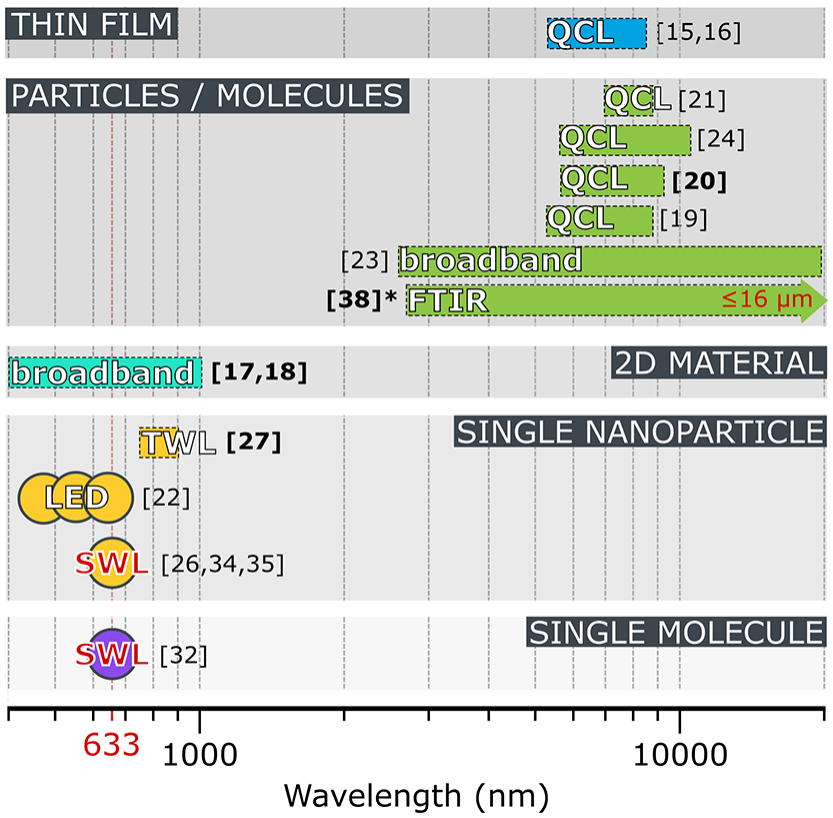
Nanomechanical resonators are a platform for spectroscopy. The collection of studies so far demonstrate their capability for measuring a broad range of substances down to single particles and molecules by photothermal spectroscopy. The bold references in brackets represent studies in the last two years; the asterisk marks an unpublished study. The only limitation of the spectral bandwidth is the absorption spectrum of the measured substance itself. This fact is evidenced by the numerous methods of illumination: a single-wavelength laser (SWL), light-emitting diode (LED), tunable wavelength laser (TWL), quantum cascade lasers (QCL), broadband light (such as from a lamp) transmitted through a monachromator, or FTIR.

**Figure 3:**
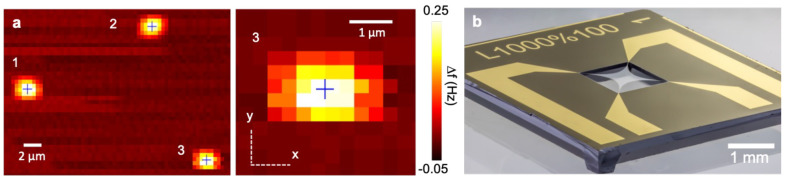
Nanomechanical resonators are a platform for microscopy. (a) Drumhead nanoresonator frequency detuning due to photothermal heating of three individual molecules (Atto633, a fluorescent dye with an attenuation cross section of 5 × 10^–20^ m^2^) from a raster scan of the excitation laser at a wavelength of 633 nm with a signal-to-noise ratio of ∼70. Reproduced with permission from ref (32), figure label changed. Copyright author(s) 2018, licensed under CC BY-NC-ND 4.0. (b) Photograph of a trampoline-shaped nanomechanical SiN resonatorFigure 4. Reproduced and cropped from ref (36). Copyright 2023 under a CC BY 4.0 license.

**Figure 4:**
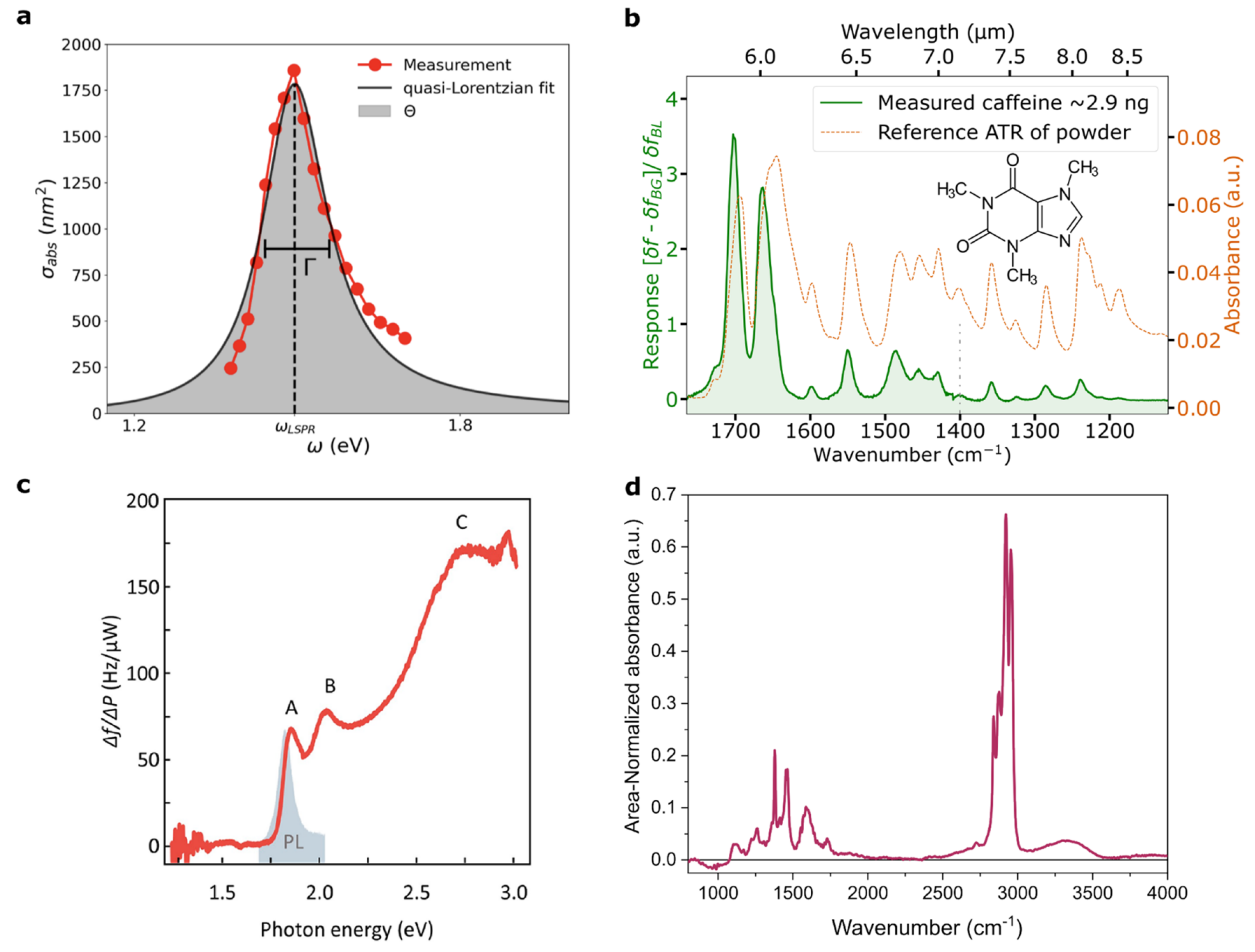
Nanomechanical photothermal absorption spectra from the most recent works demonstrating the wavelength range and variability of probing source. (a) Near-IR plasmon absorption spectrum of a 50 nm-long silica-coated Au nanorod, using a tunable-wavelength laser (TWL), reproduced from ref (27). Copyright 2023 under a CC BY 4.0 license. (b) Comparison of IR spectra of aerosol impacted caffeine by nanomechanical photothermal spectroscopy with that of the FTIR-ATR spectrum; the heating beam is a quantum cascade laser Reproduced from ref (20). Copyright 2023 under a CC BY 4.0 license. (c) Raw frequency response of a MoS2-SiN-hybrid resonator device as a function of photon energy using a TWL. Reprinted with permission from ref (17), Copyright 2022 American Chemical Society. (d) Initial findings of an estimated 124 ng of polypropylene nanoparticles absorption using a SiN trampoline resonator using the principle of FTIR. See ref (38), unpublished; figure adapted with permission from Invisible-Light Laboratories GmbH, copyright 2023. Data in part d are not intended for publication elsewhere.

Though explorations into nanoelectromechanical systems (NEMS) have led down many avenues of cutting-edge research, only tens of applications showcase their potential for photothermal spectroscopy (see Figure 2); additionally, focused light enables photothermal microscopy, localizing particles far below the diffraction limit and allowing for their characterization. Individual Atto633 molecules have been localized photothermally on SiN drumhead resonators by Chien, et. al (see Figure 3a), and their locations were corroborated with their fluorescent signatures.^32^ Employing a higher numerical aperture objective nearer the sample within the vacuum chamber, as opposed to a long working-distance objective, could have achieved resolutions akin to those in thermo-optical studies.^33^ Nonetheless, the same authors employed SiN trampolines (Figure 3b) to position 200 nm diameter spin-coated gold nanoparticles with the assistance of an atomic force microscope (AFM) tip, photothermally localized with 3 Å precision using a 633 nm laser.^34^ Progressing from these seminal studies, a very recent study utilized drumhead resonators to investigate the polarization dependence and near-IR absorption spectra of individual gold nanorods, which are ∼50 nm long with a 4:1 aspect ratio (see Figure 4a).^27,34^ Again, trampoline nanoresonators enabled also analysis of the carbon content of direct-write plasmonic Au nanostructures, verifying the corresponding enhancement of localized surface plasmon fields between two bowtie structures.^35^

Due to their high sensitivity, the nanomechanical resonator photothermal method excels in fingerprinting low-abundance species, due to its high sensitivity and resolution. Being a single-laser method, nanomechanical resonators allow for versatile adaptation across a broad spectral range. They also are an effective platform for a variety of sampling methods, as described in Sample Preparation on Nanomechanical Resonators, given the analyte can survive the low-pressure environment. However, the strength of the method extends beyond fingerprinting. Though the majority of photothermal methods are more suited for studies of analyte in liquid, despite case-by-case limitations related to the surrounding solution or matrix, the nanomechanical platform extends its observational capacity to environments typical of studies in heterogeneous catalysis and other surface science investigations.

Ventures into the infrared (IR) spectroscopy using nanomechanical structures began around the year 2013 with absorbed trace concentrations of explosive mixtures on cantilevers and strings, where the IR spectral ”fingerprints” of 42 fg (190 attomoles) of RDX were resolved.^21,37^ Around the same time, silicon nitride string resonators were utilized to capture the absorption spectrum of aerosol-sampled polyvinylpyrrolidone (PVP) particles, organic compounds coated on TiO_2_ nanoparticles, and the SiN string itself, achieving a detection limit of 44 fg with monochromatic mid-IR light.^23^ Among studies of thin films of PVP and the drug Tadalafil, membranes, irradiated with a quantum cascade laser (QCL), accomplished a signal-to-noise ratio six times higher than that of FTIR-ATR.^15,16^ For a comparison with other photothermal methods, refer to the Signal-to-Noise Ratio of the nanomechanical photothermal method discussed below.

This handful of applications indisputably demonstrates nanoresonators’ capability as direct, *in situ* spectrometers, with their wavelength range bound only by the absorption spectrum of the analyte. Recent advances in nanomechanical resonator spectroscopy have not only widened the explored wavelength range but also underscored their adaptability to new light sources, spectroscopic methods, resonator designs, and applications. In the most recent work, Kanellopulos et al. demonstrated near-IR polarization-dependent spectromicroscopy of ∼50 nm gold nanorods, as mentioned above (see Figure 4a).^27^ This effort not only yielded a high signal-to-noise ratio without a modulated heating laser, but also uncovered the potential of nanomechanical resonators for highly sensitive broadband linear, and possibly circular, dichroism spectroscopy.

Furthermore, nanomechanical photothermal spectroscopy can be used to monitor chemiphysical processes occurring on the resonator surface or the physical behavior of the resonator itself. Some examples of such observed processes are desorption and phase transitions, which may be considered ”orthogonal” to the method’s spectromicroscopic capacity.^15,39,40^ Such spectral ”eavesdropping” during thermal desorption was demonstrated in 2014, as the absorption spectra of a mere 2 fg (that is 190 attomoles) of RDX condensate was measured on silicon nitride nanostrings.^21^ A recent study by Luhmann et al. built upon this concept with thermal desorption of various mass loads of aerosol-impacted caffeine and theobromine, hyphenating NEMS-based thermal desorption with IR spectroscopy (NEMS-IR-TD).^20^ In harmony with the mass sensing capability of the nanoresonator, the authors obtained characteristic thermal programmed desorption (TPD) dynamical traces of the analyte and condensates in addition to their spectra. The spectrum of caffeine, as compared to that obtained by FTIR-ATR is shown in Figure 4b. With the aid of a Peltier element for temperature control, an analysis of time-dependent spectra during isothermal desorption allowed for separation of the spectra of species with differing desorption energies by time-resolved spectral analysis. This highlights the method’s potential use in surface and separation sciences. Along these lines, a growing body of literature is supporting the idea that the current utility of nanomechanical photothermal spectroscopy extends to studies in material physics and potentially to surface physics.

Two recent, significant contributions by Kirchhof et al. bear archetypical significance toward this point. In their first study,^17^ they obtain the *in situ* absorption spectra of 2D materials, from 400 nm to 1 μm (see Figure 4c), integrated into the resonator itself. These structures represent extended single molecules, or ∼7.2 × 10^8^ atoms, for which a measurement of the simultaneously reflected light is then corroborated to obtain the most precise, and conceivably accurate, determination of a 2D material’s dielectric function to date. This demonstrates nanomechanical photothermal spectroscopy at the fundamental limit for 2D materials, as the substance being measured *is entirely the detector of its own absorption spectrum*. Their second study demonstrates spectral measurements along with a noise-equivalent power (NEP) of 890  of silicon nitride drumhead resonators, on which the 2D materials were integrated, at room temperature.^18^ The hybrid 2D-material-resonator systems readily yielded the excitonic transition of WS_2_, plasmonic modes and intraband transitions of a plasmonic supercrystal, and dipole–dipole excited state transitions in CrPS_4_. Spectra of the latter material is said to have been attained with a mere 1 μW incident radiation, and the SiN drumhead suffers not even a 2-fold reduction in responsivity at intermediate temperatures down to 4 K.

In a broader aspect, the faculty of nanomechanical photothermal sensing is also merited by its versatility of implementation in diverse applications, which can be performed in conjunction with its photothermal capabilities. This includes its full compatibility with the Fourier transform infrared absorption spectroscopy (FTIR) technique.^38^Figure 4d shows one such preliminary result of 124 ng of aerosol-impacted polypropylene nanoparticle dispersion, with ∼50 nm diameter (Lab261 PP50), measured with NEMS photothermal spectroscopy as compared to a standard ATR-FTIR spectrum.

## Performance and Capabilities

The analyte sampled on nanomechanical photothermal detectors becomes a part of the detector system itself, coupled through thermal energy transfer. This distinguishes the nanomechanical photothermal method from other photothermal approaches, reducing the number of noise sources in the detected signal, and allowing for a variety of sampling techniques. At times there arises a need to compromise the resonator’s design for sampling over complete sensitivity optimization. For example, aerosol sampling requires a compensation in the resonator design, reducing its responsivity; nonetheless, a large signal-to-noise ratio (SNR) is achievable for such designs.^19,20^ In many applications, however, the full potential of nanomechanical resonators as spectrometers has not been fully explored.

### Fundamental Characteristics

Though the description of noise processes in mechanical resonators and circuits are well-established^8^ and adapted to nanoresonators for guiding their optimization,^9^ the interplay of such processes and their dependence on the structure’s geometry is a developing discourse.^10,12,41^ Nonetheless, an advantage of suspended nanoresonators is their endless variety of forms: strings, drumheads, trampolines, and more intricate structures, including physics-driven or topologically optimized structures such as phononic crystals and spiderwebs optimized by machine learning.^42−44^ Likewise, variations in fabrication, such as the reduction in stress by oxygen plasma tuning,^45^ and in material, such as graphene,^46^ yield resonators more responsive to thermal exchanges. A separate consideration is the resonator’s physical interaction with the incoming light. The 50-nm-thick SiN has high transmittance over most of the electromagnetic spectrum, except for a peak about 12 μm.^47^ Absorption would only raise the noise limit in that region. However, absorption by the resonator, which serves as a substrate itself, can be mitigated compared to the analyte by modifying its thickness. This adjustment permits constructive transmission for a preferred band of wavelengths.^48^ For methods hyphenated with nanomechanical photothermal spectroscopy, ultimate sensitivity is certainly not always necessary, but applications such as single-molecule spectromicroscopy call for optimization to reduce the various forms of noise.

In the nanomechanical photothermal method there are minimally two sources of noise: the single heating light source and the thermally induced noise in the resonator detection system. Most other photothermal methods require two shot-noise limited light sources, for heating and probing, and a thermally or electronically noise-limited photodetector. However, the power responsivity of the detector/analyte system, having wavelength-dependent absorptance α_*abs*_(λ) relates the relative, or fractional, frequency shift *y* = (*f*_*final*_ – *f*_*initial*_)/*f*_*initial*_ directly to the incident power *P*_0_ according to
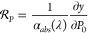
1

The caveat is that, in all resonators, higher responsivity also increases sensitivity to thermal noise. Nonetheless, research shows that nanomechanical resonators can exhibit higher signal-to-noise ratios than other photothermal methods.^27,32^

#### Signal-to-Noise Ratio

It is difficult to make a fair comparison of the nanomechanical photothermal method with other photothermal methods not only because it is not as mature as other photothermal methods. Versatility in sample type and environment, broad wavelength range, and high spatial resolution are all desirable capabilities. However, one single method does not possess all of these traits. Yet, the attributes of the nanomechanical method, such as its optical simplicity, the flexibility in resonator geometry and sampling, and notably, its elevated SNRs, position it as a valuable addition in the realm of photothermal techniques.

**Figure 5:**
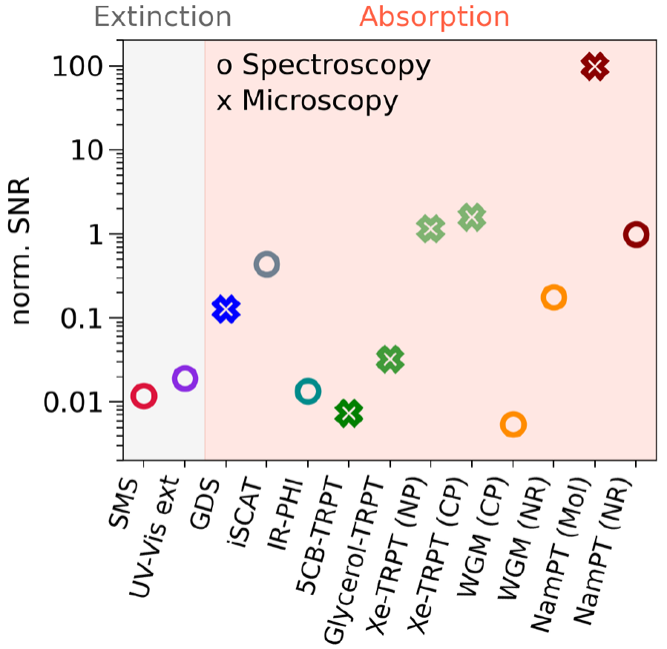
A normalized comparison of the signal-to-noise ratios of contemporary and state-of-the art single molecule/particle methods, selected, in part, due to available information to perform the calculation following the procedure in ref (27). The spectromicroscopic methods are spatial modulation spectroscopy (SMS);^49^ UV–vis extinction;^50^ ground-state depletion (GSD);^51^ interferometric scattering (iSCATT);^52^ infrared photothermal heterodyne imaging (IR-PHI);^53^ thermorefractive photothermal (TRPT) microscopy with a glycerol medium,^54^ and a nanoparticle (NP) and single conjugated polymer (CP) in supercritical xenon;^55,56^ whispering gallery mode (WGM) microscopy with a CP and nanorod (NR);^57,58^ and nanomechanical photothermal (NamPT) on a single atto633 molecule (mol) and NR.^27,32^

One possible means of comparing the available methods consists in normalizing the SNR of the various techniques, taking into account the analyte’s power absorbed and the dwell time, for the signal received. The calculation, outlined in the recent publication by Kanellopulos et. al,^27^ was performed for a handful of single-molecule and single-particle studies, whose results are shown in Figure 5. The selection of studies is resctricted by the available data provided in the referenced publications, allowing a comparison of the different state-of-the-art methods. It is worth noting that thermorefractive methods (green empty crosses) could only approach the SNR of nanomechanical resonance photothermal spectroscopy (dark red empty circle) when utilizing a supercritical xenon sample environment. The latter (NamPT study of a nanorods), though not even representing optimized conditions, surpass the collection of categories of photothermal spectroscopy represented.

**Figure 6:**
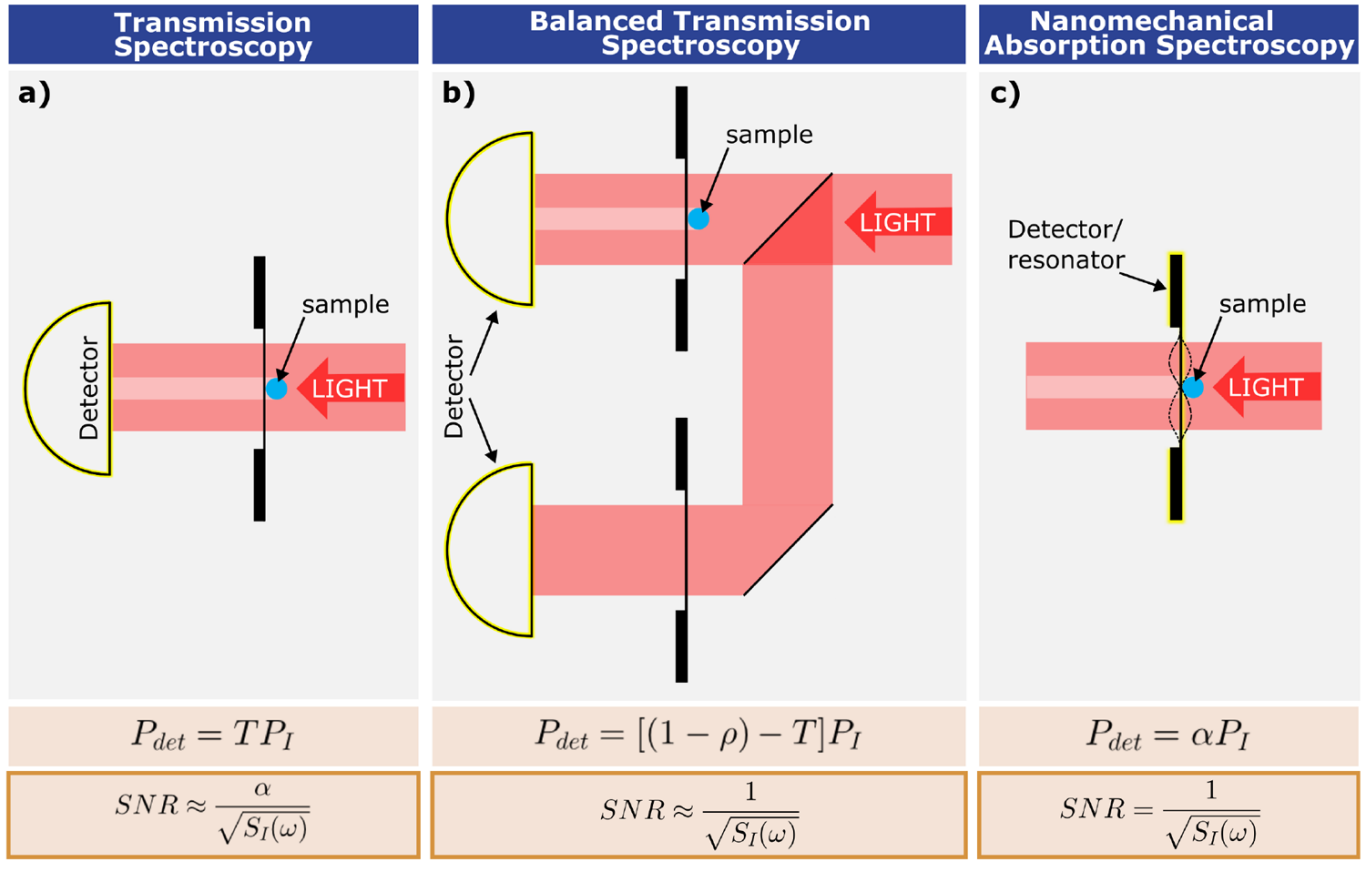
Various means of spectroscopic analysis of a sample on a thin, non-interfering substrate, using (a) single-ended transmission, (b) balanced transmission, or (c) a nanomechanical photothermal sensing scheme. Only in the case of nanomechanical absorption spectroscopy is the power detected (*P*_*det*_) directly related to the sample’s absorptance, and the SNR matches that of a balanced detection scheme in its relation to the relative intensity noise *S*_*I*_(ω).

The distinct advantage of the nanomechanical detection paradigm lies in the fact that the power absorbed by the sample is not deduced, but, rather, directly measured, and at the very least, its signal-to-noise ratio (SNR) matches that of a balanced detector (see Figure 6). To emphasize this point, consider three basic measurement techniques—transmission, balanced transmission or scattering, and nanomechanical absorption spectroscopies—assuming a gold particle that is smaller than 80 nm (below this size, the scattering cross-section becomes modestly less than its absorption).^59^ The absorptance of such a sample is very small, i.e., α = *P*_*abs*_/*P*_*I*_ ≪ 1, where *P*_*I*_ is the power of the incident light and *P*_*abs*_ the absorbed power. Assuming equal performance among the detectors, though nanomechanical detectors are only fundamentally limited by thermostatistical noise processes, as discussed in the next subsection, the underlying noise source in all three conditions is the relative intensity noise found in the probing light , expressed in terms of the relative spectral density *S*_*I*_(ω) (Hz^–1^). Furthermore, all powers are considered to be detected with the same bandwidth, and the influence of the thin substrate is omitted (that is *P*_det_, written in the schematic). The primary distinction to be underlined between these three spectroscopic techniques concerns the absorbed power detected (expressed below each schematic in Figure 6). A general expression for the SNR can be written as
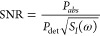
2whose value is provided for each technique in the bottom sections of Figure 6.

In transmission spectroscopy, as depicted in Figure 6a, the sample is irradiated with probing light, and the sample’s wavelength-dependent absorption is inferred from either the transmitted or the reflected light. In this scenario, reflectance is typically much smaller than transmittance *T* ≫ ρ, independent of the sensitivity of the detector that is used to measure the transmitted IR light. For a typically small absorptance α, this inevitably results in a small . The situation gets even worse for light sources with large relative intensity noise as with IR QCLs, for example, which can be suppressed with a balanced detection scheme.^60^

Balanced detection is a solution to eliminate correlated (non-quantum) intensity noise of the probing light source: the probing IR light is split into a probing and an identical reference beam where the light is not interacting with the sample itself (Figure 6b). The reference beam allows the distinction of the remaining fractional interactions with the incident light, such as reflectance (ρ), found in the background signal. While balanced transmission measurements are technically more complex than simple transmission measurements, the resulting SNR is strongly enhanced, allowing even for the detection of single-molecule absorption.^52^ Nonetheless, the SNR of a balanced detection scheme is vulnerable to quantum noise, such as shot noise.^61^ Subtracting the signal of the probing beam from the reference signal removes the signal due to the power fluctuations. As a result, the SNR improves compared to transmission spectroscopy: .

In the case of a nanomechanical photothermal measurement, as depicted in Figure 6c, the SNR is given by
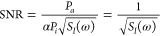
3showing that nanomechanical resonance detectors offer the same SNR and enhancement factor (the ratio of the SNR of a photothermal method to that of transmission spectroscopy),^4^ as a balanced detection scheme: *E* ≈ α^–1^.

In nanomechanical photothermal sensing, the sensitivity is solely dictated by the relative intensity noise of the light source, which can be due to thermal, electronic, or ultimately shot noise. Due to the typically long thermal time constant of nanomechanical resonators in the ms range,^11,12^ the low-frequency relative intensity noise of the light source is most relevant and should be as low as possible.

#### Noise-Equivalent Power

As nanomechanical resonators progress toward more applications in single-molecule and single particle studies, the challenge of reaching lower detection limits lies increasingly in the resonator’s own thermostatistical mechanical behavior and how much its temperature responsivity amplifies the behavior. Defining the ultimate boundaries of spectromicroscopy with these sensors requires a full understanding of their thermal, optical, and electrical properties. Naturally, this includes considerations for their design and the surrounding nanoenvironment. Consequently, research in recent years has been drawn toward elucidating the relationship of resonator material and geometry to its thermal dynamics and mechanical response.^34,42^

As the SNR describes the performance of the resonator-analyte system, the noise-equivalent power (NEP) is the bandwidth-independent performance parameter for the resonator design alone and expresses the lowest measurable heat flux, i.e., a flux of the same magnitude of the detector frequency noise. The NEP is expressed in units of W/Hz^1/2^ and is defined as^62^
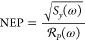
4with *S*_*y*_(ω) being the fractional frequency noise power spectral density (Hz^–1^), and  the power responsivity (eq 1, W^–1^). The detector can, therefore, be improved by minimizing the former and maximizing the latter. To gain a clearer understanding of these contributions, we need to examine the role temperature plays in them.

The power responsivity of a nanomechanical resonator represents the resonator’s intrinsic fractional frequency response to temperature change, , scaled inversely by its thermal conductance *G*:
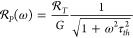
5

The radical expression represents the low-pass behavior of the resonator, dropping for power fluctuations faster than the resonator’s thermal time constant τ_*th*_ = *C*_*th*_/*G* (typically 0.1–100 ms), given heat capacity, *C*_*th*_. The temperature responsivity R_*T*_ depends on two material parameters: the temperature-induced softening, which affects Young’s Modulus (*E*), and thermal expansion, affecting the tensile stress. The former is the dominant photothermal effect found in beams, plates, and cantilevers or pillars. The latter is the responsible effect in prestressed nanoresonators: strings, drum heads, and trampolines, and variations thereof. In beams, for example,  ≈ α_*E*_/2, where α_*E*_ is the so-called thermal softening coefficient.^62^ In strings, the quantity depends, rather, on the tensile stress, σ:  ≈ α_*th*_*E*/(2σ). As α_*E*_ and α_*th*_ are typically of the same order,  for strings is enhanced by a factor of *E*/σ, much larger than unity for most materials. Therefore, the responsivity of prestressed resonators is significantly larger than that of unstressed structures, with the possibility of further optimization by stress-tuning.^32^*This makes prestressed resonators more suitable for photothermal sensing applications.*

The conductance, *G* (in the denominator of eq 5), is composed of a radiative and conductive heat transfer component, *G* = *G*_*rad*_ + *G*_*cond*_, in the absence of convection, as in the vacuum environment. Drumheads and strings are naturally suited for achieving high thermal conduction isolation due to their large aspect ratios (lateral size, or length to thickness). Instead, the radiative coupling, expressed via *G*_*rad*_ ≈ 4*A*_*s*_*εσ*_*B*_*T*^3^ for small temperature variations,^8^ can be the limiting factor for the detector’s performance. *A*_*s*_ is here the emitting surface area, σ_*B*_ the Stefan–Boltzmann constant, *T* is the temperature, and ε, the emissivity. Even in the absence of thermal conduction, the resonator still couples to the environment radiatively, defining the lowest achievable heat conductance.^12,13^ Therefore, *high photothermal sensitivity requires radiative heat transfer minimization to achieve high thermal isolation*. A possible solution is the choice of a resonator material with the very low emissivity ε. Ceramics, such as thin-film silicon nitride are excellent candidates with a low emissivity of the order of ε ≈ 0.05 for typical ∼50 nm-thin structures.^12,13^

Higher sensitivity demands, at the same time, lower noise in the measuring system (*S*_*y*_(ω) in eq 4). Assuming a low electronic readout noise detector, the relative frequency noise can be expressed as the sum of uncorrelated thermomechanical (*S*_*y*_*thm*__) and temperature fluctuation noise (*S*_*y*_*th*__):^9^
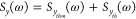
6

*S*_*y*_*thm*__ results from the thermomechanical amplitude vibration of the nanomechanical resonator driven by its own thermal energy. This amplitude noise, in turn, manifests itself in the frequency noise via amplitude-to-phase noise conversion. In the assumption of low damping (*Q* > 100), the thermomechanical noise reduces to a white noise source with the power spectral density^63^
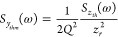
7where *Q* is the quality factor, *z*_*r*_ is the vibrational amplitude of the resonator, and *S*_*z*_*th*__ is the power spectral density of the thermomechanical displacement noise. Equation 7 shows that *the influence of thermomechanical noise can be minimized by driving the nanomechanical resonator to the maximal vibrational amplitude limit, at the onset of nonlinearity.*(64)

*S*_*y*_*th*__ comes from thermostatistical fluctuations of the resonator temperature according to the fluctuation–dissipation theorem, which directly produces frequency noise. For a lumped-element model with a concentrated mass linked to a thermal reservoir via a thermal conductance *G*, it can be simplified to^8,9^
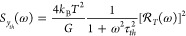
8

In the case that *G* is dominated by conductive heat transfer, the origin of *S*_*y*_*th*__ can be explained as temperature fluctuations due to the resistance in the conductive heat transfer. In the case of a resonator in the fully radiative heat transfer regime, the origin of *S*_*y*_*th*__ can be understood as temperature fluctuations created by the statistical nature of emitted and received thermal photons. *Both the thermomechanical and temperature fluctuation noise can be potentially suppressed by cooling.*(10) In the *special case* that heat transfer by conduction is negligible and heat radiation dominates (*G* ≈ *G*_*rad*_), thermal fluctuation frequency noise can dominate (*S*_*y*_*thm*__ ≪ *S*_*y*_*th*__). As a result, the NEP (4), with (5), (6), and (8), reduces to
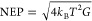
9

This expression shows that NEP can be optimized by minimizing *G* and by lowering the temperature of the resonator. However, the exact prediction of NEP remains uncertain as both the thermomechanical^11,63,65^ and temperature fluctuations^10^ remain an active subject of investigation.

#### Detection Limit of a String Resonator

In the following, the sensitivity of a string resonator is calculated, an example illustrating the power of next-generation nanomechanical photothermal sensors for single-molecule spectroscopy. Such a simple geometry has already demonstrated capability for single-particle spectroscopy.^22^ The first noise contribution, the thermomechanical fractional frequency noise (7) at resonance, for a string is found with the equipartition theorem to be^62^
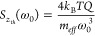
10

Here, *m*_*eff*_ is the effective mass of the resonator with the eigenfrequency  for a specific mode *n*. *L* is the string length and ρ, its mass density.

As discussed above, *S*_*y*_*thm*__ can be minimized by maximizing the coherent vibrational amplitude *z*_*r*_ of the resonator, governed by its geometric nonlinearity:^66^
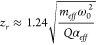
11where α_*eff*_ is the effective Duffing nonlinearity parameter, which for a string of cross-sectional area *A* is given by α_*eff*_ = (*nπ*)^4^EA/(8*L*^3^).^62^

The second noise contribution (8) scales inversely with the thermal conductance. Since thermal fluctuations happen all along the length of the string,^8,9^ an effective conductance *G*_cond_^*^ can be derived by averaging the thermal resistance over the entire string length *L*
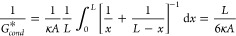
12where κ is the thermal conductivity of the string’s material. For the conductance due to thermal radiation (*G*_rad_^*^), both the top and bottom surfaces of the string are accounted for, and a linear temperature field is assumed,^62^ giving a the total effective thermal conductance as
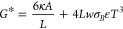
13where *w* is the string width.

**Figure 7:**
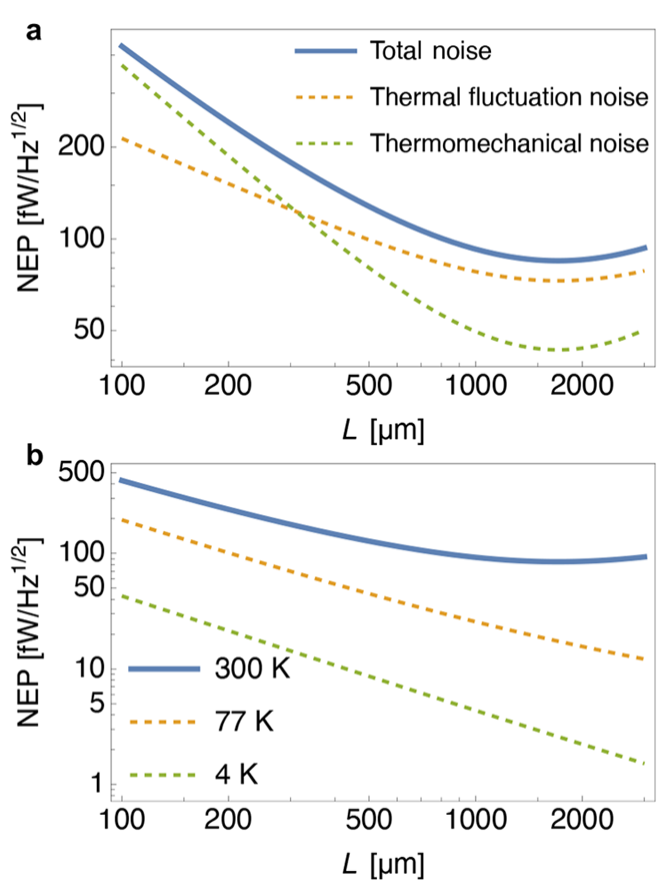
Noise equivalent power (4) calculated for the fundamental mode of a 50 nm thick and 1 μm wide SiN string resonator for various lengths, based upon the interplay of the two limiting thermal noise mechanisms (a) and the profiles for resonators cooled by liquid hydrogen and helium compared to room temperature (b). The plots are based on the following parameters: ρ = 2700 kg/m^3^, *E* = 250 GPa, κ = 3W/(mK), α = 2.2 ppm/K, and ϵ_*rad*_ = 0.05.

An interplay of these two phenomena contributing to the NEP, with differing string lengths, is modeled for typical SiN 50-nm-thick resonators in Figure 7a. It can be seen that for longer strings, at room temperature the thermomechanical noise is not ubiquitously the primary noise source, as often suspected.^65^ In this regime, thermal fluctuations constitute the frequency noise limit with a minimal NEP ≈ 100 fW/Hz^1/2^ at room temperature. The temperature plays a major role in this sensitivity as well, yielding NEP values of just a few femtowatt at ∼4 K for longer strings (see Figure 7b). Reducing the temperature, in this way, is the only foreseeable means of overcoming the blackbody limit in these detection systems. The thermal fluctuation noise, with its quadratic temperature dependence, is negligible below room temperature, positioning thermomechanical noise as the limiting factor.

In conclusion, the inherent thermally induced fluctuation constraints of nanoscale resonators delineate the unparalleled sensitivities of NEMS resonators to temperature-dependent changes in stress. Further improvements rely upon reducing the factors which govern these fundamental fluctuation noise sources. Moreover, the performance of dynamic measurements in the future will inherently require striking a balance between the speed of the resonator and its sensitivity, as these two factors are inversely proportional.

### Sample Preparation on Nanomechanical Resonators

**Figure 8:**
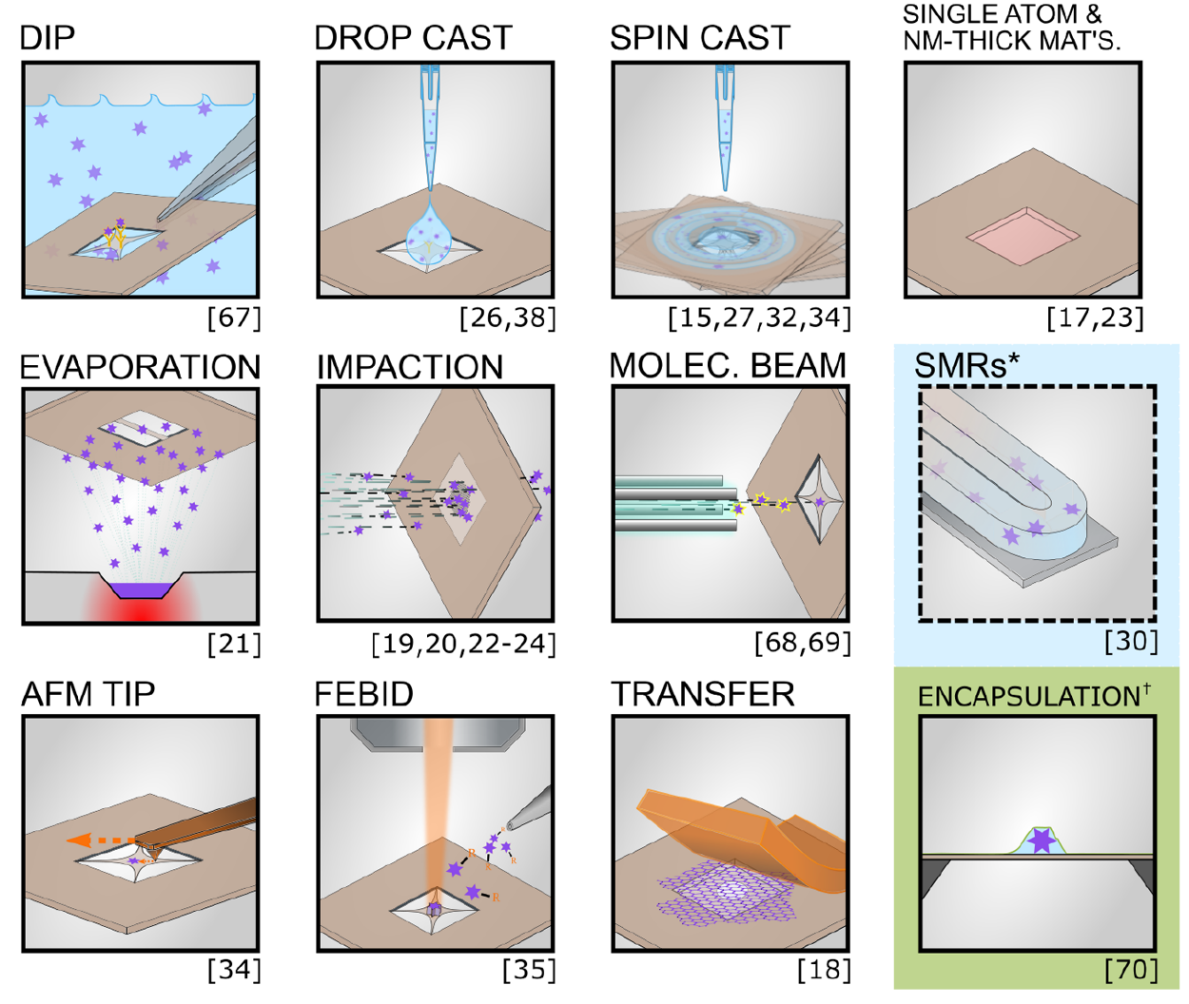
Nanomechanical resonators are a platform for direct sampling in several mass detection, spectroscopic, and localization studies. (∗) Suspended michrochannel resonators (SMRs) rely upon the same state-of-the-art fabrication technologies as nanoresonators. (†) Encapsulation of hydrated samples with graphene in vacuum conditions on 50-nm-thick SiN has been demonstrated yet remains untested in nanomechanics.^70^

Nanometer- and subnanometer-thick suspended structures facilitate a variety of sampling methods already employed in chemistry, life sciences, and surface science and materials. Figure 8 is an illustrative synopsis of the use of these structures as direct-sampling platforms in various applications to date. Though many of these applications do not involve spectroscopy, the resonator takes on the role of a substrate, which can subsequently be used for detection. Fabricated in their diced wafer section, or chip, the resonators can be functionalized, passivated,^20^ or left bare, then dipped into a liquid suspension in the same way that microstructure detectors have been.^67^ Analyte can also be drop-casted or spin-coated onto the resonator-chip.^15,26,27,32,34^ Single nanoparticles, molecules, and thin films have been detected following these basic sampling procedures, and spin-coated nanoparticles can be subsequently positioned with the aid of the cantilever tip of an atomic force microscope (AFM).^34^ For airborne analyte, nebulized solutions, or suspensions, aerosol impaction is the method of choice.^19,20,23,24^ In vacuum, suspended nanostructures are suitable for adsorption of evaporated analyte or that produced by electrospray ionization and subsequently directed or focused into a molecular beam as in mass spectrometry.^68,69^ For studies of the physical properties of materials, transfer by stamping or even focused electron-beam induced deposition can be considered a means of sampling.^17,18,35^

Nanomechanical photothermal spectroscopy can, therefore, be applied to a vast range of substance distributions, from thin films to single molecules. The majority of these cases allow for pressures just below 10^–3^ mbar, which is achievable within minutes using a roughing pump. Though the analyte in these studies are in solid phase upon detection, an exception is found in suspended microchannel resonators (SMRs), microresonators with nanometer-thick walled channels hold pico- to femtoliters of solution (highlighted in Figure 8).

Fascinatingly, Arble et. al have demonstrated encapsulation of hydrated samples, even living cells, by graphene on 50-nm-thick SiN windows while in vacuum.^70^ Although it is uncertain how such windows would perform at resonance, successful implementation of this approach would have profound implications for photothermal spectroscopy by nanomechanical resonators.

## Future Applications

From thin films, to surfaces, down to single molecule spectromicroscopy, nanomechanical resonators possess wide-ranging capability in the process of being explored. Immediate applications include characterization of 2D materials^17,18^ and various plasmonic structures.^26,27,35^ Considering nanomechanical resonators can operate in UHV and at cryogenic temperatures potentially places these detectors at the forefront of other fields of research. Though applications in cutting-edge heterogeneous catalysis, for example, and quantum systems are on the horizon, the study of functional nanomaterials will continue to be consequential, a springboard into these fields. Even interactions on materials in thermal contact with the resonator, such as thin layers or nanoparticles, may also be observed. Principally, this method is susceptible to all forms of spectroscopy, which can be performed in free-space.

### Circular Dichroism Spectromicroscopy

As the polarization-dependence has already been shown for single gold nanorods,^27,34^ one immediate example is circular dichroism spectroscopy limited to surface-adsorbed molecules or particles, especially relevant to pharmacology. It is important to note that much has been accomplished toward photothermal dichroism spectroscopy and microscopy employing the thermo-optical effect. These advances are laid out nicely in a recent review by Adhikari and Orrit, including a discussion of the challenges of measuring single nano-objects.^2^ Both nanomechanical and thermorefractive methods would be negatively affected by the depolarizing tendencies of high numerical aperture objectives. Where a soft matrix or liquid are not essential to measure and characterize the analyte, the nanomechancial resonance detuning method would likely be superior as there would be no concern for mixing of dark-field and bright-field scattering of the surrounding medium, as it is strictly a direct absorption measurement of the analyte. Circular dichroism spectroscopy is just one of many applications clearly within our reach, outlining a rich and exciting future for nanomechanical absorption spectroscopy.

### Analysis of Functional Nanomaterials

Functional nanomaterials, such as luminescent nanoparticles and nanoscale catalysts, are some of the most consequential advancements of nanotechnology. Quantum dots, for instance, have risen to prominence, becoming integral in various imaging, sensing, and photonic applications. Additionally, metal nanoparticles and nanoclusters offer enhanced catalytic efficiency and open new avenues for in-depth exploration in the dynamic field of heterogeneous catalysis.

On the single-particle level, the heterogeneity discussed in the Introduction is a crucial concern in fundamental research related to materials chemistry and surface science. In particular, for atomically precise metal nanoclusters, differences of a single atom significantly affect catalytic reaction mechanisms and optical properties.^71^ Furthermore, it is often challenging to scale up nanomaterial synthesis, while characterization of such materials is only possible on the scale of a single particle.

Single-particle microscopy, such as atomic-force microscopy or electron microscopy, has been crucial in understanding the heterogeneity of nanoparticles and has facilitated functional materials development.^72^ Likewise, specific optical properties of single nanoparticles are a vital feature in many applications. In the case of metal species, optical properties are strictly related to particle size and structure, and the corresponding absorption bands, generally found in the UV–vis range, typically shift to lower wavelengths as particle size reduces.^73,74^ However, standard UV–vis spectroscopy only allows the measurement of a particle ensemble, necessitating the sample’s separation and purification using methods like size-exclusion chromatography (SEC).^75^ On the other hand, nanomechanical photothermal spectroscopy is capable of enabling the distinction between individual particles’ distinctive features and provides direct information about sample heterogeneity without separation steps. The method allows for spectroscopy across the full spectral range, where IR in combination with UV–vis absorption can be used to gain chemical information on a nanomaterial, monitor ligand exchange reactions, provided there is sufficient sample material^76^ and the samples do not ionize, and investigate luminescent nanoparticles as well as chiral nanomaterials.^77^

The comparison of the highly fluorescent Atto 633,^32^ with a signal-to-noise ratio of 70, to other single molecules and particles in terms of attenuation coefficients, cross sections, and heat dissipation ratios is outlined in Table 1. The single-particle sensitivity will allow the characterization of precious, individually fabricated nanomaterials, which are often difficult to fabricate. Nanoresonator spectrometers serve as a robust platform for the investigation of individual thermoplasmonic and dipole–dipole interactions along with their dependencies. Such interactions play a pivotal role in a myriad of applications, ranging from medicine to light harvesting, due to their profound influence on functionality.^78^ Metallic nanoparticles themselves have been crucial instruments in the spectroscopy of attached or neighboring analyte and have demonstrated enhancement of absorption in several solar cell configurations.^79^ Plasmonic-enhanced spectroscopy on a thermally sensitive nanoresonator, however, will enable plasmon-enhanced absorption spectroscopy of even nonfluorescing trace substances and single molecules, improving the signal-to-noise ratio and allowing for investigations into their spectral dependencies on surface interactions.

**Table 1:** List of Molar Attenuation Coefficients and Cross Sections of Selected Samples at Application-Relevant Wavelengths in the UV-Vis and IR^a^

	Spectral wavelength	Molar attenuation coefficient ε [M^–1^ cm^–1^]	Atten. cross- section *A* [m^2^]	Heat dissipation ratio β	ref
Hepatitis B virus protein	UV (280 nm)	7,320,000	2.8 × 10^–18^	∼0.8	(80)
4 nm Au nanoparticle	Vis (506 nm)	3,600,000	1.4 × 10^–18^	1	(81)
BSA protein	IR (6 μ m)	190,000	7.3 × 10^–20^	1	(82)
Atto 633	Vis (633 nm)	130,000	5.0 × 10^–20^	0.38	(32)
BSA protein	UV (280 nm)	44,000	1.7 × 10^–20^	∼0.8	(83)
Lysozyme protein	UV (280 nm)	38,000	1.5 × 10^–20^	∼0.8	(83)
Lysozyme protein	IR (6 μ m)	36,000	1.4 × 10^–20^	1	(82)
Hepatitis B virus monomer	UV (280 nm)	30,500	1.2 × 10^–20^	∼0.8	(80)
Virus RNA nucleotide	UV (260 nm)	8,000	3.0 × 10^–21^	∼1	(80)
Insulin protein	UV (280 nm)	6,000	2.3 × 10^–21^	∼0.8	(83)
Tryptophan (amino acid)	UV (280 nm)	5,500	2.1 × 10^–21^	0.8	(83)
Virus RNA nucleotide	UV (280 nm)	4,000	1.5 × 10^–21^	∼1	(80)
Tyrosine (amino acid)	UV (280 nm)	1,490	5.7 × 10^–22^	0.86	(83)
Single peptide bond	IR (6 μ m)	312	1.2 × 10^–22^	1.0	(84)
Cysteine (amino acid)	UV (280 nm)	125	4.8 × 10^–23^	N/A	(83)

a The heat dissipation ratio for proteins^85^ and nucleotides^86^ in the UV has been estimated from their quantum yield. The molar attenuation coefficient and cross-section are directly linked by *A* = 3.82 × 10^–27^ε.

### Analysis of Single Biomolecules

In the single-molecule sensitivity regime, a sample can be identified by way of its individual specific absorption coefficient. The amount of light a sample species attenuates at a specific wavelength is determined by its molecular composition, and can be represented by its molar attenuation coefficient ε. In proteins, UV light’s attenuation at ∼280 nm can primarily be attributed to the three amino acids tryptophan (Trp), tyrosine (Tyr), and cysteine (Cys). For Beer–Lambert-type measurements of proteins in solution, a proteins’ molar attenuation coefficient can be estimated from the sum of the number of individual contributions of these three amino acids^83^
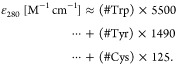
14

The molar attenuation coefficient is highly specific to the amino acid composition of an individual protein, and thus it is most probably identifiable in solution. However, toward far lower concentrations, a similar method could attempted for single proteins to give, at least, some probablility for differentiating species. Ideally, the UV absorption from a single protein can be used as a molecule-specific fingerprint, much in the same way the mid-IR spectral fingerprint is used to identify a myriad of molecular species.

IR bands not only serve an identification function, but a quantification function for proteins. It has been shown that the attenuation signal at the amide I band (6 μm) correlates strongly with the amino acid count of a protein.^87^ The amide I signal is created by the sum of all peptide bonds that link consecutive amino acids. Therefore, from the amide I attenuation of an individual protein, it is possible to estimate the number of amino acids and identify the protein.

Concerted effort is being directed toward applications such as proteomics to find alternatives for protein identification with improved sensitivity.^88^ Acquiring chemical fingerprints, in addition to mass, allows for a multiphysical analysis, which provides an enhanced knowledge base for the identification and characterization of individual proteins and protein complexes. Here, nanomechanical single-molecule UV–vis combines with single-molecule IR absorption spectroscopy to create a promising new method for protein identification and biomolecular analysis, in general.

### Fingerprinting and Separation Science

The published limits, ranging from pg to fg for nonfluorescent analyte, underscore the heightened sensitivity inherent to nanomechanical photothermal spectroscopy. Fundamentally, measurement of such trace amounts can be accomplished with unfocused light, which minimally requires a single optical element, attesting to the simplicity of its implementation. Despite operating exclusively in low-pressure environments, nanomechanical resonators are inherently apt for surface science applications, having demonstrated efficacy in analyzing and monitoring thin films and their phase transitions.^16,89^ Furthermore, spectral ”eavesdropping” can foreseeably reveal information about a molecule’s interactions with the surface of the resonator or vicariously through a nanoparticle on its surface. This includes monitoring of isomeric transitions and binding affinities of trace substances down to the single molecule level, adding valuable insight into surface reaction processes. The method might also facilitate surface-assisted laser desorption of analyte,^90,91^ whether adsorbed directly onto the resonant material or another layer on its surface. This would allow the monitoring of individual chemical groups of a species spectrally during isothermal desorption under the influence of narrow linewidth IR light, for instance. When operated at visible wavelengths, utilizing scanning focused light could potentially enable direct surface sampling mass spectrometry imaging on the resonator.^91^ After all, the method provides time-dependent spectral data in harmony with mass exchange, which carries with it valuable information regarding trends in desorption energies, as demonstrated in Luhmann et al.^20^ This study further shows that in mixtures, it is possible to distinguish and identify species through singular value decomposition or potentially through global spectro-temporal and principal component analyses during isothermal desorption. This avenue of application leaves much yet to be explored but reveals a unique direction for separation science, where temperature control and chemical composition of the surface can be adapted for the separation of diverse analyte mixtures.

## Challenges

However, as with many technologies at the cutting edge of research, real-world implementation remains the primary challenge in regard to commercial and industrial applications for trace substance identification and quality control. The primary challenge lies in the impediments of vacuum systems: they are bulky and have some pump-down time and venting time. Nonetheless, the compromise between processing time and sensitivity is often inconsequential, as evidenced when comparing nanomechanical IR absorption spectroscopy of thin films to ATR-FTIR measurements.^24^ At other times, conventional methods introduce artifacts in the spectra which are not seen in nanomechanical photothermal spectroscopy, and the vacuum environment will be a necessary trade-off, especially at the single-molecular level.^15^ Nonetheless, for trace substance analysis, with an apt system for sample exchange, the speed at which measurements are performed can be improved. Alternatively, at the expense of optimal sensitivity due to ballistic losses, many first-stage pumps can reach an ultimate partial pressure of 1 order of magnitude beyond ∼10^–3^ mbar in air, which can be reached in a few minutes.^23^ At these pressures, however, a stabilized vacuum or a compensation mechanism for frequency drift is required. Despite this, recent research has shown that 10^–1^ mbar has low-noise performance for *in-plane* modes of doubly clamped beams.^92^

With subnanometer localization resolution at visible wavelengths, nanomechanical photothermal spectroscopy shows potential to resolve the whole range of particulate matter, which threatens global health and the ecological environment,^93^ and to spectromectrically distinguish between surface-adsorbed particulates.^94^ This requires effective and accurate sampling mechanisms and sample-to-measurement tracking. Such accounting is necessary for all cases where native concentrations are desired, as analyte can be lost either in the process of sampling on resonator chips or between this and the measurement process. In low-pressure environments, where temperature control cannot be leveraged to minimize analyte loss, rates of desorption post pump-down can serve to approximate near-original concentrations on the resonator.^20,39^ Additionally, the low-pressure environment is not conducive to studies in solution, yet SMRs, though challenging to fabricate, have already been used for single protein gravimetric detection and nJ absorbed power sensitivity.^30,31^ Though single protein spectroscopy would be a significant challenge for SMRs and likely require attachment to a metallic nanoparticle for photothermal enhacement, it is unlikely, with their mass, that anything smaller could be investigated.

However, graphene encapsulation of analyte, even living cells, in their liquid environment was developed some years ago for scanning electron microscope studies on coverglass substrates.^95^ More recently graphene encapsulation has been accomplished on silicon nitride windows, projected to be a viable means for spectromicroscopy for hydrated samples in the future.^70^ Demonstrating photothermal spectroscopy of molecules in an encapsulated wet environment would be groundbreaking, expanding the nanomechanical photothermal method to new areas of chemistry and life sciences. Should the nanomechanical resonators maintain their high signal-to-noise ratios, encapsulation would give this method a distinct advantage over other photothermal methods.

## Conclusions

Nanomechanical photothermal spectroscopy enables the measurement of thermal relaxation in substances, down to a single molecule, where the studied substance becomes an integral component of the detection mechanism. The adaptability in fabrication, design, sampling, and probing light characteristics of nanoresonators is broadening the scope of state-of-the-art photothermal spectroscopy and merits further explorations into trace species identification, single-entity characterization, and numerous, real-time, simultaneous or hyphenated studies. To date, studies have demonstrated that thin ceramic and 2D material resonators are capable of photothermally probing by various light sources and techniques: from electronic absorption of a single particle by directed, incoherent light to focused light with subdiffraction-limited localization of nanoparticles and even FTIR of highly dilute nebulized, aerosol-impacted solutions. Geometry and surface structure variability, along with the optical simplicity, makes this method highly adaptable. Despite the necessity for *in vacuo* measurements, it does not translate to a sacrifice in throughput due to pump-down and venting times in all cases. Furthermore, the thermostatistically limited sensitivity of nanoresonators allows for a higher signal-to-noise ratio than many other photothermal methods, with room for further optimization. There is also room for more cutting edge applications in low-pressure environments, such as surface sciences and heterogeneous catalysis. As nanomechanical photothermal spectroscopy is inevitably leading toward more applications in single-molecule spectroscopy, there is, likewise, room for increased sensitivity where 2D materials and phononic crystals are concerned. There are also unexplored concepts, such as graphene encapsulation on nanomechanical resonators, which shows great potential for studies with hydrated samples. Nonetheless, in the gap between the single molecule limit and the current state-of-the-art, there remains a wealth of ground-breaking applications waiting to be explored and perfected.
