# Correction to “Photothermal Microscopy and Spectroscopy with Nanomechanical Resonators”

**DOI:** 10.1021/acs.jpcc.3c08019

**Published:** 2024-01-03

**Authors:** Robert G. West, Kostas Kanellopulos, Silvan Schmid

We have uncovered a misleading paragraph in the latest perspective article, on page 21921, which we would like to clarify. Just above the subsection “Noise-Equivalent Power”, it is stated at the conclusion of the discussion of the signal-to-noise ratio (under “Performance and Capabilities” and “Fundamental Characteristics”) that “In nanomechanical photothermal sensing, the sensitivity is solely dictated by the relative intensity noise of the light source”, as expressed in eq 3.

This is *only true* when the sensitivity of the nanomechanical resonator is sufficient to resolve the intensity fluctuations of the light source. On the one hand, to fully benefit from the improved SNR of nanomechanical resonators, they need to be sensitive enough to resolve the intensity fluctuations of the light source , where NEP is the noise equivalent power with units of [W/Hz^1/2^]. On the other hand, if the sensitivity is too low to resolve the intensity fluctuations, that is , the signal-to-noise ratio is given by
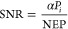
1

The discussion of the NEP in the following section of the paper is also then justified: reducing the NEP leads the resonators to the limit of the intensity fluctuations of the light source.

